# Greater Glycaemic Responses to Bodyweight Change in South Asians Than White Europeans at High Risk of Type 2 Diabetes

**DOI:** 10.1111/dom.70945

**Published:** 2026-06-01

**Authors:** Franciskos Arsenyadis, Joseph Henson, Pratik Choudhary, Melanie J. Davies, Kamlesh Khunti, Tom Yates

**Affiliations:** ^1^ Diabetes Research Centre University of Leicester Leicester UK; ^2^ NIHR Leicester Biomedical Research Centre University Hospitals of Leicester NHS Trust and University of Leicester Leicester UK; ^3^ NIHR Applied Research Collaboration East Midlands Leicester UK

**Keywords:** cohort study, glycaemic control, insulin resistance, type 2 diabetes, weight management

## Abstract

**Aims:**

Bodyweight change is an important determinant of cardiometabolic health, but its metabolic impact may differ by ethnicity. This study investigated whether ethnicity (White European, South Asian) modifies the association between change in bodyweight and changes in HbA1c and oral glucose tolerance test (OGTT)–derived measures.

**Materials and Methods:**

Cohort data were analysed from the UK PROPELS trial (ISRCTN:83465245), which recruited adults at high risk of type 2 diabetes. Bodyweight and metabolic biomarkers were measured at baseline, 1‐ and 4‐years. Trial arms were analysed as a single cohort. Generalised estimating equation models examined interactions between ethnicity and change in bodyweight with changes in HbA1c and OGTT‐derived measures, adjusting for randomisation group, age, sex, socioeconomic factors, smoking, medication use and baseline values.

**Results:**

A total of 543 participants with ≥ 2 bodyweight and OGTT observations were included (371 White European; 172 South Asian). Each 5 kg change in bodyweight was associated with a greater change in HbA1c in South Asians (1.5 mmol/mol, 95% CI: 1.0–2.0) than White Europeans (0.9 mmol/mol, 0.6–1.3; *p* for ethnicity interaction = 0.034). A significant ethnicity interaction was also observed for 2‐h post‐challenge glucose (*p* = 0.003), with larger changes in South Asians (0.9 mmol/L) than White Europeans (0.4 mmol/L). No ethnicity interactions were observed for fasting glucose or insulin measures.

**Conclusions:**

Bodyweight changes have a greater adverse impact on glycaemic control in South Asian populations at high risk of type 2 diabetes, driven by stronger associations with post‐challenge glucose, highlighting the potential risks and benefits of modest bodyweight perturbations.

## 1 Introduction

Excess adiposity adversely impacts glycaemic control and is a known driver of type 2 diabetes [1]. Ethnicity has an impact on glycaemic control [2] with certain ethnic groups, particularly South Asians (SAs), exhibiting a significantly higher risk of developing type 2 diabetes at a lower bodyweight‐for‐height ratio (i.e., BMI) compared to White Europeans (WEs). SA ethnicity is a broad term referring to populations originating from countries of the SA region—including Afghanistan, Bangladesh, Bhutan, India, Maldives, Nepal, Pakistan and Sri Lanka. In England, the vast majority of the SA diaspora is of Indian, Pakistani or Bangladeshi origin [3]. SAs develop type 2 diabetes earlier [4, 5] and experience higher overall prevalence rates [6].

While bodyweight changes modify risk of type 2 diabetes in all ethnic groups [1], it remains unclear how markers of glycaemic control, such as HbA1c, fasting glucose or post‐challenge insulin sensitivity, respond to bodyweight changes across different ethnic groups (i.e., SAs compared to WEs).

Meta‐analyses of lifestyle interventions indicate that even modest sustained reductions in bodyweight (< 5 kg) can be effective in reducing risk of type 2 diabetes across ethnic groups [7]. Ethnicity‐specific differences in glycaemic control may not be captured by HbA1c alone, which reflects chronic glycaemia but is insensitive to early changes in insulin resistance and beta‐cell function [8]. Fasting glucose can remain within the normal range for many years due to compensatory fasting hyperinsulinaemia [9]. Measuring fasting insulin alongside glucose provides insight into hepatic insulin resistance, while post‐challenge measures capture glucose tolerance and peripheral insulin sensitivity. Together, these provide a more comprehensive metabolic profile [9].

SAs typically exhibit higher fasting and post‐challenge insulin concentrations, reflecting compensatory beta‐cell responses to underlying insulin resistance [10]. However, sustained metabolic demand can place increased stress on beta‐cell function over time, potentially contributing to earlier beta‐cell failure, particularly in SA populations who may have reduced beta‐cell reserve [10, 11], suggesting earlier beta cell dysfunction and accelerated decline in insulin sensitivity [10]. These patterns can be detected through the oral glucose tolerance test (OGTT), in which glucose and insulin are measured following ingestion of a standardised oral glucose load, enabling identification of early disturbances in glucose metabolism that fasting glucose and insulin alone may miss. As such, OGTT‐derived measures offer insight into insulin resistance and beta‐cell function by capturing the dynamic insulin response needed to maintain glucose homeostasis.

Previous studies using repeated OGTT have demonstrated ethnic differences in insulin dynamics, showing that SAs experience a more rapid decline in insulin sensitivity and earlier beta cell exhaustion compared to WEs [12, 13]. Furthermore, SAs often present with higher circulating insulin levels for the same glucose concentration, consistent with greater insulin resistance, alongside higher HbA1c levels, which may reflect differences in postprandial glucose excursions and overall glycaemic exposure [14, 15, 16]. As these differences are detectable in the thin‐fat phenotype described in SA neonates [17], this suggests that underlying metabolic differences may manifest early in life and contribute to the increased risk of type 2 diabetes. However, despite these insights, studies exploring ethnic differences in the association between changes in bodyweight and repeated OGTT‐derived measures, such as fasting and post‐challenge glucose and insulin concentrations, are limited. Quantifying these differences may help to better contextualise ethnicity‐specific risk associated with adverse bodyweight changes. The aim of this study was to investigate whether associations between changes in bodyweight and HbA1c or OGTT‐derived markers of fasting and post‐challenge glucose metabolism differed between SA and WE adults at high risk of type 2 diabetes in the UK.

## 2 Materials and Methods

This secondary analysis reports data from the PRomotion Of Physical activity through structured Education with differing Levels of ongoing Support for people at high risk of type 2 diabetes (PROPELS) trial (ISRCTN83465245). The protocol and main trial results have previously been reported in detail [18, 19]. Briefly, PROPELS was a 4‐year (recruitment period: 2013–2015) multi‐site (Leicester and Cambridge, England) randomised controlled trial recruiting individuals at high risk of developing type 2 diabetes who were randomised (stratified by sex and ethnicity) to either: (a) a control group receiving a detailed information leaflet about knowledge and perceptions of diabetes risk and the importance of physical activity, (b) an intervention group receiving the Walking Away programme of structured physical activity support which included a three‐hour group‐based behavioural intervention designed to address knowledge and perceptions of risk factors for type 2 diabetes and the effectiveness of physical activity in managing those risks, along with pedometers to support goal setting and self‐monitoring or (c) the Walking Away programme with additional text support. Ethical approval was granted by the National Health Service (NHS) National Research Ethics Committee, Leicester (04/05/2012; ref.: 12/EM/0151). All participants provided written informed consent and the study was carried out in accordance with the principles of the Declaration of Helsinki 2013 and adhered to the regulations for Good Clinical Practice.

### 2.1 Cohort

Eligible participants were aged 40–74 years (25 to 74 years if SA), recruited across two trial sites with a confirmed plasma glucose or HbA1c in the non‐diabetic hyperglycaemia range within the last 5 years. Non‐diabetic hyperglycaemia was defined as fasting glucose ≥ 5.5 mmol/L and < 7.0 mmol/L, 2‐h post‐challenge glucose ≥ 7.8 mmol/L and < 11.1 mmol/L or HbA1c ≥ 6.0% (42 mmol/mol) and < 6.5% (48 mmol/mol) [18]. Data were collected during researcher‐led clinic visits at Baseline, Year 1 and Year 4.

### 2.2 Exposures

Ethnicity was reported through participant self‐declaration during researcher‐led interview, consistent with standard data capture practice in UK health research and clinical datasets. Participants self‐reporting as White British, White Irish or Any other White background were grouped into the WE cohort, while those reporting Indian, Bangladeshi, Pakistani or Any Other Asian background were grouped into the SA cohort. For the purposes of this analysis, only participants self‐reporting as WE or SA according to the above definitions were included.

Bodyweight was measured to the nearest 0.1 kg and obtained through calibrated scales (Tanita, West Drayton, UK). Height was measured by stadiometer to the nearest 0.5 cm and BMI was calculated by dividing bodyweight by the square of height.

### 2.3 Covariates

Data on demographics (age, sex, Indices of Multiple Deprivation [IMD]) were collected at baseline. Each participant's residential area was assigned an IMD score to reflect their socioeconomic status. IMD scores are publicly available, continuous indicators of overall social and material deprivation, derived from a range of factors including income, employment, education, and housing. The scores were stratified into quintiles (1 indicating high deprivation and 5 low deprivation). Occupation type (retired, sedentary, standing, manual, and heavy manual), smoking (never, ex‐smoker, current), statin and blood pressure medication use were collected by researcher‐led interview.

Daily step count, valid wear time (in minutes and days) data were derived from a waist‐worn accelerometer (Actigraph GT3X) with up to 7 days reported at each timepoint [15, 18]. Data were recorded at 100 Hz and aggregated into 60‐s epochs for analysis. Non‐wear time was defined as at least 60 consecutive minutes of zero counts, and a valid day required a minimum of 600 min of wear time [18]. Participants were included in the analysis if they had at least three valid days. Accelerometer data were processed using a commercially available software tool (KineSoft version 3.3.76, Kinesoft, Loughborough, UK; www.kinesoft.org).

### 2.4 Outcomes

HbA1c values were obtained through venous sampling, collected into a 2.7 mL fluoride tube and processed through hospital pathology services under local standard operating procedures conforming to external quality assurance reference values. Any detected type 2 diabetes cases based on HbA1c values were excluded from progressing to randomisation.

A sub‐cohort (Leicester site only) undertook OGTT at Baseline, Year 1 and 4. Participants arrived fasted to the research centre (Leicester Diabetes Centre, UK) in the morning and were seated before venous blood samples of glucose and insulin were obtained. Participants then ingested a glucose load (75 g carbohydrate, 410mls Lucozade) and a further blood sample of glucose and insulin was obtained at 120 min. Plasma glucose and insulin samples were subsequently stored at −80°C and batch analysed at the end of the study period using commercially available assays for insulin (Biotechne, USA, insulin intraplate variation 4.0%) and Yellow Springs Institute 2500 Biochemistry Analyser (Xylem, USA) for glucose.

HOMA‐IR was obtained through OGTT fasting parameters, defined as follows:

$$\begin{array}{c} (\text{Fasting Plasma Glucose}\;\left[\text{mmol}/L\right]\;\\ x\;\text{Fasting Plasma Insulin}\;\left[\mathrm{m\mu}/L\right])/22.5 \end{array}$$

HOMA‐IR offers a method used to estimate the degree of insulin resistance [20]. Higher HOMA‐IR values indicate greater insulin resistance. Values > 2.0 are usually indicative of insulin resistance, but how this varies based on ethnicity is not clear.

Matsuda Index was obtained through OGTT fasting and post‐challenge parameters defined as follows:

$$\begin{array}{c} 10,000/\surd \left[\text{fasting glucose}\;\left(\text{mmol}/L\right)\times \text{fasting insulin}\;\left(\text{pmol}/L\right)\right]\\ \times \left[\text{mean glucose}\;\left(\frac{\text{mmol}}{L}\right)\times \text{mean insulin}\;\left(\frac{\text{pmol}}{L}\right)\right] \end{array}$$

Matsuda Index offers a method used to estimate the degree of insulin sensitivity [21]. Higher Matsuda Index values indicate better insulin sensitivity.

### 2.5 Statistical Analysis

Generalised Estimating Equations (GEE) linear models (assumptions of normality verified) with an exchangeable correlation matrix were used to examine the association between changes in bodyweight and concurrent changes in measures of glycaemia and insulin sensitivity and the interaction with ethnicity across two levels (Baseline to Year 1, Year 1–4). The dependent variables were defined as changes in outcome measures between consecutive timepoints (Baseline to Year 1 and Year 1–4), with corresponding change in bodyweight as the primary independent variable. Each participant contributed up to two observations corresponding to these intervals (Baseline to Year 1 and Year 1–4). Models were additionally adjusted for the baseline values of each level for both outcome (change in HbA1c or OGTT‐derived measure) and exposure variable (change in bodyweight) in addition to identified covariates (sex, age, ethnicity, randomisation group, IMD, employment type, smoking, statin or blood pressure medication status). These models were repeated to investigate interactions between bodyweight change and timepoint or randomisation group and a separate sensitivity analysis was undertaken which excluded SAs outside the age criteria for WEs (< 40 years). Finally, models were further adjusted for accelerometer wear time and change in step count to investigate whether associations were confounded by changes to physical activity levels.

Beta‐coefficients are presented per 5 kg change in body weight to aid clinical interpretability, with 95% CI. *p*‐values of < 0.05 were accepted as statistically significant. In the GEE models, β‐coefficients represent associations between changes in bodyweight and HbA1c or OGTT‐derived measures and may be interpreted symmetrically; for example, a positive coefficient indicates that changes in both variables occur in the same direction, whether increasing or decreasing. Analysis was performed using SPSS version 29 (IBM Corp. 2023).

## 3 Results

PROPELS included 1366 participants of which 910 (66.6%) were recruited from the Leicester site. Of these, 750 (82.4%) had at least 2 bodyweight observations and, of these, 543 had at least 2 OGTT observations. As anthropometric and health outcomes were largely unaltered in all randomised groups apart from small differences between groups in bodyweight (~1 kg) at 12‐ and 48‐month follow‐up [19], the trial population was pooled and analysed as a single cohort. The robustness of this approach was tested statistically by including an interaction term between randomisation group and change in bodyweight or between timepoint and change in bodyweight in the below modelling to assess whether randomisation group or timepoint modified the results; in all cases, the interactions were not significant (*p* > 0.05) (Table S1).

Of the 543 participants included in this sub‐analysis, 371 were WE (49.9% female) and 172 were SA (44.2% female). These 543 participants contributed 994 change observations (WE = 671, SA = 323), corresponding to up to two intervals per participant (Baseline to Year 1, and Year 1–4), and formed the OGTT cohort. Table 1 describes the characteristics of PROPELS participants included in the GEE model at baseline and Table 2 describes the unadjusted, adjusted and age‐sensitivity modelling for HbA1c and OGTT‐derived outcomes.

**TABLE 1:** Baseline demographics, anthropometric and clinical characteristics of participants included in the GEE model.

Baseline characteristics	White European (*n* = 371)	South Asian (*n* = 172)
Age (years)	62.3 ± 8.1	55.1 ± 9.9
Sex	Male = 186 Female = 185	Male = 96 Female = 76
Weight (kg)	84 ± 18.2	74 ± 15.8
Body Mass Index (kg/m^2^)	29.9 ± 5.4	27.5 ± 4.8
HbA1c (mmol/mol)	39.2 ± 3.5	39.7 ± 3.4
HbA1c (%)	5.7 ± 0.3	5.8 ± 0.3
Statin status	Yes = 125 (33.7%) No = 246 (66.3%)	Yes = 45 (26.2%) No = 127 (73.8%)
Blood pressure medication status	Yes = 169 (45.6%) No = 202 (54.5%)	Yes = 53 (30.8%) No = 119 (69.2%)
Occupation (type)	Sedentary = 61 (16.4%) Standing = 33 (8.9%) Manual = 6 (1.6%) Retired/Other = 271 (73.1%)	Sedentary = 42 (24.4%) Standing = 18 (10.5%) Manual = 3 (1.7%) Retired/Other = 109 (63.4%)
Smoking status	Never smoked = 179 (48.3%) Ex‐smoker = 159 (42.9%) Current smoker = 33 (8.9%)	Never smoked = 129 (75.0%) Ex‐smoker = 29 (16.9%) Current smoker = 14 (8.1%)
Indices of Multiple Deprivation (quintile)	3.2 ± 1.4	2.5 ± 1.2
Valid wear days	6.7 ± 0.8	6.7 ± 0.7
Daily step count	6939.1 ± 2982.5	7074.8 ± 3135.8
OGTT‐derived measures		
Fasting glucose (mmol/L)	5.9 ± 0.8	5.8 ± 0.8
Post‐challenge glucose (mmol/L)	7.2 ± 2.5	6.9 ± 2.3
Fasting insulin (mIU/L)	11.1 ± 7	13.1 ± 2.3
Post‐challenge insulin (mIU/L)	63.9 ± 52	96.7 ± 82
HOMA‐IR	0.50 ± 0.34	0.57 ± 0.39
Matsuda Index	592.8 ± 454.7	485.4 ± 431.4

*Note:* ± Denotes standard deviation.

**TABLE 2:** Unadjusted and adjusted GEE modelling results for change in metabolic outcomes per kg change in bodyweight.

Outcome			Unadjusted(*n* = 543, 994 observations)		Adjusted(*n* = 543, 994 observations)		Adjusted + physical activity (*n* = 527, 934 observations)	
			Change inoutcome	Ethnicity interaction	Change inoutcome	Ethnicity interaction	Change in outcome	Ethnicity interaction
HbA1c (mmol/mol)	South Asian	Change in bodyweight (per 5 kg)	**1.71 (1.22 to 2.19)** ***p* < 0.001**	***p* = 0.024**	**1.46 (0.96 to 1.96)** ***p* < 0.001**	***p* = 0.034**	**1.35 (0.87 to 1.84)** ***p* < 0.001**	*p* = 0.050
	White European		**0.97 (0.60 to 1.34)** ***p* < 0.001**		**0.94 (0.60 to 1.27)** ***p* < 0.001**		**0.91 (0.58 to 1.24)** ***p* < 0.001**	
Fasting glucose (mmol/L)	South Asian		**0.21 (0.05 to 0.38)** ***p* = 0.011**	*p* = 0.245	**0.17 (0.02 to 0.32)** ***p* = 0.024**	*p* = 0.358	**0.15 (0.00 to 0.30)** ***p* = 0.048**	*p* = 0.689
	White European		**0.11 (0.01 to 0.20)** ***p* = 0.031**		**0.11 (0.02 to 0.20) *p* = 0.018**		**0.10 (0.01 to 0.19)** ***p* = 0.041**	
Fasting insulin (mIU/L)	South Asian		**3.17 (0.47 to 5.86)** ***p* = 0.021**	*p* = 0.514	**3.70 (0.76 to 6.65)** ***p* = 0.014**	*p* = 0.390	2.70 (−0.23 to 5.63) *p* = 0.071	*p* = 0.759
	White European		**2.22 (1.27 to 3.17)** ***p* < 0.001**		**2.25 (1.27 to 3.23)** ***p* < 0.001**		**2.13 (1.19 to 3.07)** ***p* < 0.001**	
2‐h glucose (mmol/L)	South Asian		**1.06 (0.63 to 1.49)** ***p* < 0.001**	***p* = 0.009**	**0.92 (0.53 to 1.32)** ***p* < 0.001**	***p* = 0.003**	**0.94 (0.53 to 1.36)** ***p* < 0.001**	***p* = 0.013**
	White European		**0.42 (0.24 to 0.61)** ***p* < 0.001**		**0.35 (0.17 to 0.53)** ***p* < 0.001**		**0.31 (0.14 to 0.47)** ***p* < 0.001**	
2‐h insulin (mIU/L)	South Asian		**14.4 (1.4 to 27.5)** ***p* = 0.030**	*p* = 0.322	9.1 (−1.7 to 19.9) *p* = 0.097	*p* = 0.650	**12.4 (0.9 to 23.8)** ***p* = 0.034**	*p* = 0.802
	White European		**7.9 (3.3 to 12.4)** ***p* < 0.001**		**7.8 (3.4 to 12.3)** ***p* < 0.001**		**6.7 (10.3 to 68.2)** ***p* < 0.001**	
HOMA‐IR	South Asian		**0.15 (0.9 to 0.21)** ***p* < 0.001**	***p* = 0.047**	**0.17 (0.09 to 0.25)** ***p* < 0.001**	*p* = 0.160	**0.13 (0.06 to 0.19)** ***p* < 0.001**	*p* = 0.352
	White European		**0.10 (0.05 to 0.12)** ***p* < 0.001**		**0.08 (0.05 to 0.12)** ***p* < 0.001**		**0.08 (0.05 to 0.12)** ***p* < 0.001**	
Matsuda index	South Asian		**−126.8 (−181.1 to −72.5)** ***p* < 0.001**	*p* = 0.294	**−105.0 (−155.9 to −54.1)** ***p* < 0.001**	*p* = 0.372	**−106.8 (−168.5 to −45.1)** ***p* < 0.001**	*p* = 0.594
	White European		**−84.1 (−128.0 to −40.5)** ***p* < 0.001**		**−75.4 (−115.1 to −35.6)** ***p* < 0.001**		**−86.7 (−127.6 to −45.8)** ***p* < 0.001**	

*Note:* Models were adjusted for randomisation group, sex, age, occupation type, deprivation through Indices of Multiple Deprivation, smoking status, statin or blood pressure medication use, baseline bodyweight and baseline metabolic values. Models were further adjusted for baseline and change to daily steps and accelerometer wear time. Results are presented with *β* (95% Confidence Interval) and *p*‐value (*p* < 0.05 in bold).

SAs were younger (55.1 vs. 62.3 years for WE), had a lower BMI (27.5 kg/m^2^ vs. 29.9 kg/m^2^) and lower IMD score (higher deprivation) relative to WEs. SAs were also less likely to be on blood pressure (30.8% vs. 45.5%) or statin medications (26.1% vs. 33.7%). SAs had higher markers of insulin resistance and poorer markers of insulin sensitivity at baseline, which was explained by higher levels of fasting and post‐challenge insulin.

Both ethnic groups were, on average, weight stable overall during the 4‐year observation period and there was no statistically significant difference between the changes in both groups for bodyweight, HbA1c, HOMA‐IR or Matsuda Index (*p* > 0.05) (Table S2).

In the primary adjusted model, each 5 kg change in bodyweight was associated with a 1.46 (95% CI: 0.96 to 1.96) mmol/mol (*p* < 0.001) change in HbA1c in SAs, compared to a 0.94 (0.60–1.17) mmol/mol (*p* < 0.001) change in WEs; *p* = 0.034 for ethnicity interaction (Table 2). Associations between bodyweight change and HbA1c were similar in unadjusted and adjusted models (SA: unadjusted: *β* = 1.71, 95% CI: 1.22–2.19, vs. adjusted *β* = 1.46, 0.96–1.96; WE: *β* = 0.97, 0.60–1.34, vs. adjusted *β* = 0.94, 0.60–1.27), suggesting that baseline differences did not materially influence the findings (Table 2). The association between change in bodyweight and change in HbA1c was linear across tertiles of bodyweight change (Figure S1).

**FIGURE 1:**
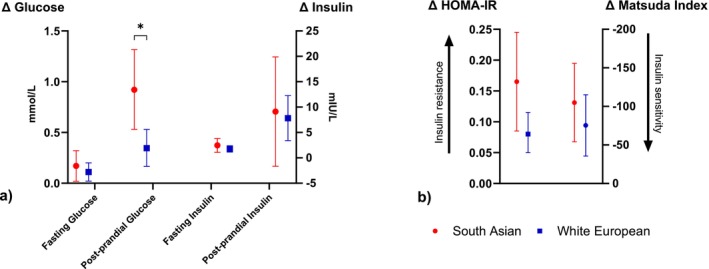
Changes to fasting and post challenge glucose and insulin (a) and OGTT‐derives measures of insulin resistance/sensitivity (b) per 5 kg weight change in South Asian and White European adults. *Ethnicity interaction (*p* = 0.003).

A significant interaction between ethnicity and bodyweight change was also observed for the association with 2‐h post‐challenge glucose (*p* = 0.003). Each 5 kg change in bodyweight was associated with a 0.92 (0.53–1.32) mmol/L (*p* < 0.001) change in 2‐h post‐challenge glucose in SAs, compared to a 0.35 (0.17–0.53) mmol/L (*p* < 0.001) change in WEs in the adjusted model. Conversely, although changes in bodyweight were associated with changes in fasting glucose, fasting insulin, and 2‐h post‐challenge insulin in both ethnicities, the magnitude of these associations did not differ between groups (Figure 1a; Table 2). While associations between bodyweight change and summarised measures of insulin resistance (HOMA‐IR) and sensitivity (Matsuda Index) were stronger in SAs compared to WEs, these differences did not reach statistical significance (Figure 1b; Table 2).

Further adjustment for physical activity did not materially alter the observed associations, though the ethnicity interaction for change in bodyweight and HbA1c did become borderline (*p* = 0.050) (Table 2), potentially due to a smaller cohort reporting physical activity metrics (*n* = 527 and 934 observations vs. *n* = 543 and 994 observations in the full cohort). Similar consistency was observed for HOMA‐IR (SAs: *β* = 0.17 [0.09–0.25] vs. 0.08 [0.05–0.12]) and Matsuda Index (SAs: *β* = −75.4 [−155.9 to −54.1] vs. −75.4 [−115.1 to −35.6]). Effect estimates remained directionally stronger in SAs (Table 2). The sensitivity analysis which excluded SAs outside the age criteria for WEs (*n* = 12 contributing 17 change observations removed) did not change model outputs (Table S3).

## 4 Discussion

In this prospective cohort study of WEs and SAs at high risk of type 2 diabetes, ethnicity significantly modified the association between bodyweight change and HbA1c, with a stronger effect observed in SAs. Each 5 kg change in bodyweight was associated with a 1.46 mmol/mol change in HbA1c in SAs compared to a 0.94 mmol/mol change in WEs over the 4‐year follow‐up period. This difference appeared to be primarily driven by a stronger association between bodyweight change and 2‐h post‐challenge glucose levels in SAs. In contrast, associations between bodyweight change and other glycaemic markers (i.e., fasting and 2‐h post‐challenge measures of insulin resistance and beta‐cell function) were comparable across ethnicities.

These results broadly agree with published literature for those at high risk of type 2 diabetes which shows that increase in bodyweight is associated with modest rise in HbA1c [1], with larger effects observed for post‐challenge glucose [22, 23]. This study extends these previous findings by demonstrating that the magnitude of these associations is modified by ethnicity. Specifically, SAs exhibited a more pronounced glycaemic response to bodyweight gain (or loss), with each 5 kg change in bodyweight associated with a clinically meaningful 0.92 mmol/L change in 2‐h post‐challenge glucose, more than double the 0.35 mmol/L corresponding change observed in WEs. At baseline, SAs had higher fasting and post‐challenge insulin compared to WEs, despite similar fasting and post‐challenge glucose levels. Changes in bodyweight were associated with changes in HOMA‐IR and Matsuda Index in both ethnic groups; however, the magnitude of these associations was relatively weak and did not differ by ethnicity, suggesting that differential changes in insulin resistance did not explain the larger post‐challenge glycaemic response in SAs. This pronounced difference in post‐challenge glucose response could instead reflect differences in peripheral glucose disposal. SAs tend to have lower appendicular muscle mass [24] and greater ectopic fat (including intermuscular fat) even at lower BMI levels [25, 26], features associated with reduced peripheral insulin sensitivity and increased reliance on beta‐cell compensation [10]. Because SAs may have inherently lower beta‐cell function [27], they may experience earlier beta‐cell exhaustion, limiting their ability to maintain glucose homeostasis with weight gain and contributing to a greater post‐challenge glycaemic response than that observed in WEs.

However, this study did not directly measure ectopic fat or insulin sensitivity (e.g., via hyperinsulinaemic euglycaemic clamps with glucose tracers). Therefore, the use of total bodyweight change alone may overlook compositional differences at baseline and follow‐up that could partly explain the observed ethnic disparities in glycaemic response.

Of note, our results are bidirectional, indicating that along with interpretations for bodyweight gain, our findings also suggest relatively modest reductions in bodyweight may be sufficient to elicit improvements in HbA1c and post‐challenge glucose among SAs. This is an important finding, given that both HbA1c and impaired glucose tolerance are well established predictors of cardiovascular mortality [28]. These results suggest that relatively modest reductions in bodyweight are associated with larger improvements in HbA1c and post‐challenge glucose among SAs and could contribute to the delay or prevention of type 2 diabetes.

This bidirectional interpretation is supported by experimental evidence from mechanistic studies. The GlasVEGAS overfeeding study demonstrated that intentional modest bodyweight gain of approximately 5 kg over 4–6 weeks resulted in greater adverse metabolic responses in SA compared with WE men, suggesting reduced metabolic buffering to bodyweight gain in this population [29]. However, in contrast to the present study, ethnic differences in response to overfeeding were largely observed in measures of insulin resistance rather than post‐challenge glucose, which may be related to the younger and healthy population in the GlasVEGAS with potential for greater beta‐cell compensation. An 8‐day study of SA and WE men undertaking very low calorie diet (VLCD) (resulting in approximately 5% bodyweight loss) showed that hepatic insulin sensitivity improved similarly in both groups, while SAs, despite higher insulin levels and lower baseline glucose disposal, demonstrated greater improvement in glucose disposal rate, primarily via nonoxidative glucose disposal [30], providing further potential mechanistic insight into the findings for glycaemia in the present study.

Taken together, these studies provide experimental and mechanistic insights that support our findings that even modest bidirectional changes in bodyweight may elicit disproportionately greater metabolic responses in SA populations. From a clinical perspective, our longer‐term observational findings reinforce the potential importance of timely and sustained lifestyle interventions that achieve modest bodyweight loss in SA populations, which may be sufficient to confer meaningful metabolic benefit even at lower BMI thresholds. Our findings also support the importance of early identification and monitoring of dysglycaemia in SA populations, particularly using measures sensitive to postprandial glucose excursions such as the OGTT.

A key strength of this analysis lies in the use of a relatively large, well phenotyped, prospective, multi‐ethnic cohort with repeated measures of both HbA1c and OGTT glucose and insulin variables, all obtained under standardised experimental conditions. Another notable strength is the 4‐year follow up, allowing for some insight into the sustained impact of modest bodyweight change on metabolic homeostasis. However, there are also some notable limitations. This analysis relied on data from the PROPELS trial where bodyweight and OGTT‐derived measures were secondary outcomes. As this was a secondary analysis, the study was not specifically powered to detect ethnic interactions, and some analyses may therefore be underpowered to detect modest differences. The PROPELS cohort was characterised by a higher baseline BMI, as participants were selected based on elevated type 2 diabetes risk, potentially limiting the generalisability of these findings to broader populations. Furthermore, SAs in the PROPELS cohort were purposefully younger (to account for the higher type 2 diabetes risk) but also had other baseline differences compared to the WE cohort, such as a higher deprivation level and a lower BMI. Although accounted for in the model, differences in age, deprivation and BMI may have led to residual confounding. However, we undertook sensitivity analysis restricting the age range of the SA cohort and found similar results to the primary model. We were unable to determine the precise timing of bodyweight change within each interval, or the duration for which participants were exposed to a given level of bodyweight gain. As such, the analysis captures average changes between study visits rather than short‐term temporal dynamics of bodyweight change and glycaemic response. There was no recording of 1‐h post‐challenge glucose which is a strong predictor of future type 2 diabetes and cardiovascular disease risk [31, 32, 33], and would have allowed for a more nuanced understanding of the relationship between bodyweight change and the glucose response curve. Though the causal effect of bodyweight change on insulin resistance and diabetes prevention is well established, as this was a cohort study the interaction by ethnicity cannot be assumed to be causal and may have been confounded by other unmeasured variables. For example, we did not adjust for ethnic differences in diet or cultural culinary practices [34]. Dietary patterns differ between SA and WE populations; however, these patterns are heterogeneous, particularly within SA diaspora groups, where there is increasing adoption of westernised dietary patterns and exposure to ultra‐processed energy‐dense foods [35]. Dietary intake was not assessed in this study, and bodyweight changes likely reflect the integrated effects of diet, physical activity, and other behavioural factors. The reliance on surrogate measures of insulin resistance and sensitivity, without immediate post‐challenge insulin and c‐peptide samples measurements limited the ability to comprehensively characterise the insulin response to a glucose challenge. Finally, we did not attempt these analyses in other ethnic groups due to low representation.

In this high‐risk population, changes in bodyweight were more strongly associated with HbA1c among SAs compared to WEs, with key ethnic differences observed in 2‐h post‐challenge glucose. These differences were not observed for indices of insulin resistance or sensitivity, indicating that the ethnic disparity in glycaemic response is primarily driven by post‐challenge glucose rather than changes in insulin resistance. These findings align with existing evidence of the heightened susceptibility to type 2 diabetes in SA populations but further identify that smaller bodyweight changes may have disproportionate positive or negative effects on metabolic health in SAs compared to WEs, which could act to modify progression to type 2 diabetes.

## Funding

Franciskos Arsenyadis was supported by the Wellcome Trust [223512/Z/21/Z] through the Leicestershire Healthcare Inequalities Improvement Doctoral Training Programme Fellowship. Kamlesh Khunti is supported by the National Institute for Health Research (NIHR) Applied Research Collaboration East Midlands (ARC EM), NIHR Global Research Centre for Multiple Long‐Term Conditions, NIHR Cross NIHR Collaboration for Multiple Long‐Term Conditions, NIHR Leicester Biomedical Research Centre (BRC), and the British Heart Foundation (BHF) Centre of Excellence.

## Conflicts of Interest

P.C. has acted as consultant for Cambridge Mechatronics Ltd., consultant, advisory board member and speaker for Medtronic, Novo Nordisk, Sanofi, Lilly, Abbott, Insulet, AstraZeneca, Dexcom, Glooko and Roche. Advisory board member for Ypsomed, Embecta and Vertex. He has received research support from Medtronic, Abbott, Novo Nordisk, Dexcom and Insulet. K.K. has acted as a consultant, speaker or received grants for investigator‐initiated studies for Abbott, Astra Zeneca, Bayer, Novo Nordisk, Sanofi‐Aventis, Servier, Lilly and Merck Sharp & Dohme, Boehringer Ingelheim, Oramed Pharmaceuticals, Pfizer, Roche, Daiichi‐Sankyo, Applied Therapeutics, Embecta and Nestle Health Science. M.J.D. has acted as a consultant/advisor and speaker for Eli Lilly, Novo Nordisk and Sanofi, has attended advisory boards for AbbVie, Amgen, AstraZeneca, Biomea Fusion, Carmot/Roche, Daewoong Pharmaceutical, Sanofi, Zealand Pharma, Regeneron, GSK and EktaH and as a speaker for AstraZeneca, Boehringer Ingelheim and Zuellig Pharma. She has received grants from AstraZeneca, Boehringer Ingelheim and Novo Nordisk. T.Y. has received funding from Astra Zeneca for an investigator‐initiated project. F.A. declares no conflicts of interest.

## Supporting information


**Figure S1:** Change in HbA1c (mean, 95% CI) across tertiles of bodyweight change (median, minimum and maximum value) across ethnic groups.
**Table S1:** GEE modelling interactions for bodyweight change and randomisation group or timepoint.
**Table S2:** Independent sample t‐tests for changes in bodyweight, HbA1c, HOMA‐IR and Matsuda Index between South Asian and White European cohorts.
**Table S3:** GEE modelling results adjusted for White European age inclusion criteria.

## Data Availability

Pseudonymised subject level data is available on a case‐by‐case basis on reasonable request. Researchers interested in accessing the data are asked to contact the principal investigator (Prof. Kamlesh Kunti; kk22@le.ac.uk).
