# Supplementary material for: Greater Glycaemic Responses to Bodyweight Change in South Asians Than White Europeans at High Risk of Type 2 Diabetes
Source: Diabetes Obes Metab. 2026 Jun 1;28(8):7291–9. doi: 10.1111/dom.70945 (PMC13341349; doi:10.1111/dom.70945)
Supplement: Supplementary file 1 — Figure S1: Change in HbA1c (mean, 95% CI) across tertiles of bodyweight change (median, minimum and maximum value) across ethnic groups. Table S1: GEE modelling interactions for bodyweight change and randomisation group or timepoint. Table S2: Independent sample t‐tests for changes in bodyweight, HbA1c, HOMA‐IR and Matsuda Index between South Asian and White European cohorts. Table S3: GEE modelling results adjusted for White European age inclusion criteria. [file DOM-28-7291-s001.docx]

| **Electronic supplementary material** |  |
| --- | --- |
| **Electronic supplementary figures** |  |
| **ESM Fig 1.** Change in HbA1c (mean, 95% CI) across tertiles of bodyweight change (median, minimum and maximum value) across ethnic groups | Page 2 |
| **Electronic supplementary tables** |  |
| **ESM Table 1.** GEE modelling interactions for bodyweight change and randomisation group or timepoint | Page 3 |
| **ESM Table 2.** Independent sample t-tests for changes in bodyweight, HbA1c, HOMA-IR and Matsuda Index between South Asian and White European cohorts | Page 4 |
| **ESM Table 3.** GEE modelling results adjusted for White European age inclusion criteria | Page 5-6 |
|  |  |

**ESM Figure 1.** Change in HbA1c (mean, 95% CI) across tertiles of bodyweight change (median, minimum and maximum value) across ethnic groups

**ESM Table 1.** GEE modelling interactions for bodyweight change and randomisation group or timepoint

| Outcome | p-value for randomisation group * change in bodyweight interaction | p-value for timepoint * change in bodyweight interaction |
| --- | --- | --- |
| HbA1c  (mmol/mol) | p=0.934 | p=0.926 |
| Fasting glucose  (mmol/L) | p=0.608 | p=0.970 |
| Fasting insulin  (mIU/L) | p=0.271 | p=0.916 |
| 2-hour glucose  (mmol/L) | p=0.702 | p=0.408 |
| 2-hour insulin  (mIU/L) | p=0.798 | p=0.625 |
| HOMA-IR | p=0.281 | p=0.757 |
| Matsuda index | p=0.267 | p=0.390 |

**ESM Table 2**. Independent sample t-tests for changes in bodyweight, HbA1c, HOMA-IR and Matsuda Index between South Asian and White European cohorts

|  | **South Asian** | **White European** | p-value |
| --- | --- | --- | --- |
| Bodyweight (kg) change | 0.2 ± 3 | 0.3 ± 4.8 | 0.125 |
| HbA1c (mmol/mol) change | 1.1 ± 2.5 | 1 ± 2.5 | 0.848 |
| HOMA-IR change | 0.08 ± 0.38 | 0.07 ± 0.34 | 0.555 |
| Matsuda Index change | -27.9 ± 320 | -31.4 ± 381.2 | 0.899 |

Data are mean and ± denotes standard deviation.

**ESM Table 3**. GEE modelling results adjusted for White European age inclusion criteria

| Outcome | | | Adjusted  (age-sensitivity)  (N=531, 977 observations) | |
| --- | --- | --- | --- | --- |
|  |  |  | Change in outcome | p-value for ethnicity interaction |
| HbA1c  (mmol/mol) | South Asian | Change  in bodyweight (per 5kg) | **0.29**  **(0.19 to 0.39)**  **p<0.001** | **p=0.028** |
|  | White European |  | **0.19**  **(0.12 to 0.25)**  **p<0.001** |  |
| Fasting glucose  (mmol/L) | South Asian |  | **0.04**  **(0.01 to 0.07)**  **p=0.019** | p=0.306 |
|  | White European |  | **0.02**  **(0.00 to 0.04)**  **p=0.015** |  |
| Fasting insulin  (mIU/L) | South Asian |  | 0.46  (-0.11 to 1.04)  p=0.117 | p=0.817 |
|  | White European |  | **0.43**  **(0.24 to 0.61) p<0.001** |  |
| 2-hour glucose  (mmol/L) | South Asian |  | **0.20**  **(0.11 to 0.28)**  **p<0.001** | **p=0.009** |
|  | White European |  | **0.07**  **(0.03 to 0.11)**  **p<0.001** |  |
| 2-hour insulin  (mIU/L) | South Asian |  | 2.0  (-0.3 to 4.2)  p=0.086 | p=0.625 |
|  | White European |  | **1.6**  **(0.7 to 2.5)**  **p<0.001** |  |
| HOMA-IR | South Asian |  | **0.03**  **(0.02 to 0.05)**  **p<0.001** | p=0.114 |
|  | White European |  | **0.02**  **(0.01 to 0.02) p<0.001** |  |
| Matsuda index | South Asian |  | **-19.6**  **(-30.2 to -9.0)**  **p<0.001** | p=0.630 |
|  | White European |  | **-15.1**  **(-23.0 to -7.1)**  **p<0.001** |  |

Model were adjusted for randomisation group, ethnicity, sex, age, occupation type, deprivation through Indices of Multiple Deprivation, smoking status, statin or blood pressure medication use, baseline bodyweight and baseline metabolic values. For this age-sensitivity analysis, data excludes South Asians <40 years, n = 12 removed (contributing 17 observations to the model). Results are presented with β (95% Confidence Interval) and p-value.
